# Correlates of sexual health service use amongst unmarried young adults in Kathmandu, Nepal

**DOI:** 10.3389/frph.2025.1587770

**Published:** 2025-10-29

**Authors:** Joshua Jayasinghe, Connie M. Ulrich, Anju Shrestha, Mamata Sherpa Awasthi, Jesse Chittams, Bridgette M. Rice, Prakash Shrestha, Anne M. Teitelman

**Affiliations:** ^1^ College of Nursing, Thomas Jefferson University, Philadelphia, PA, United States; ^2^ Biobehavioral Department, School of Nursing, University of Pennsylvania, Philadelphia, PA, United States; ^3^ Department of Medical Ethics and Health Policy, Perelman School of Medicine, University of Pennsylvania, Philadelphia, PA, United States; ^4^ Emergency Department, Royal Prince Alfred Hospital, Camperdown, NSW, Australia; ^5^ School of Population Health, Curtin University, Perth, WA, Australia; ^6^ M. Louise Fitzpatrick College of Nursing, Villanova University, Villanova, PA, United States; ^7^ School of Nursing, University of Pennsylvania, Philadelphia, PA, United States

**Keywords:** sexual health services, Nepal, health services research, sexual violence, youth friendly health services (YFHS)

## Abstract

**Background:**

In Nepal, sexual relationships outside of the traditional arranged marital system are increasingly common. Despite the availability of modern sexual health care, research on how to effectively deliver sexual health services to unmarried young adults in Nepal is limited.

**Objective:**

This study examined key theoretical correlates between Andersen's Behavioral Model of Health Service Use and actual sexual health service use among unmarried young adults (aged 18 to 25) in Kathmandu, Nepal.

**Methods:**

Unmarried young adults between 18 and 25 were recruited from colleges and universities in the Kathmandu area. A total of 110 women and 93 men completed the survey (*n* = 203). Using a cross-sectional correlational design, the analysis involved descriptive statistics, bivariate analysis, and logistic regression.

**Findings and conclusions:**

Approximately 37% of participants reported engaging in sexual intercourse, with 55.7% reporting condom use during most recent intercourse. Less than half (39.1%) were aware of available sexual health services, and less than one-third (26.6%) were aware of Human Papillomavirus (HPV). Over a third of participants (40.9%) reported experiencing unwanted sexual contact, and 5.9% had a history of forced sexual intercourse. The actual reported sexual health service use within the past 12 months was 13.9%. Logistic regression analyses showed higher perceived youth friendliness of the health system (OR: 1.19; CI: 1.01–1.39; *p* < .05), sexual attraction to the same or both sexes (OR: 2.91; 95%; CI: 1.54–5.50; *p* < .01), higher perceived sexual risk (OR: 1.33; 95%; CI: 1.11–1.59; *p* < .01), as well as prior health awareness and behaviors (e.g., cigarette consumption, dating app use), were statistically significant correlates of sexual health service use. These findings indicate a need for further research to understand the experiences of Nepalese young adults with sexual healthcare and essential elements of youth-friendly health systems.

## Introduction

Based on United Nations data from 2019, Nepal has one of the highest adolescent birth rates for young people ages 15 to 19 (63 per 1,000 girls) amongst all South Asian Association for Regional Cooperation (SAARC) countries (1). Nepal also has a lower rate of satisfying the demand for family planning (58%) compared to the median (65%) in SAARC countries. The maternal mortality ratio is higher in Nepal (186 per 100,000) compared to the overall SAARC median (178 per 100,000). Factors contributing to maternal mortality in Nepal include early marriage, long distances to reach medical care, lack of family planning, unsafe abortion care, and lack of quality healthcare services and midwives (2, 3).

While Nepal has made progress in expanding healthcare and family planning services, access to modern methods of family planning and other sexual health services remains uneven, leaving young adults in Nepal particularly vulnerable to poor sexual health outcomes. These outcomes include new HIV infections, increased risk for maternal mortality attributed to unsafe abortion, unwanted pregnancies, and a high adolescent birth rate (4). Limited research in Nepal has found that young people (ages 18–30) lack access to appropriate and adequate sexual health services, underscoring the need for further research on how to deliver these health services effectively (5–8).

While Nepal has traditionally had an arranged marriage system, research suggests that sexual relationships before marriage and casual relationships have become more common, especially in urban areas. A survey of emergency contraceptive use among 185 women in Pokhara, Nepal found that 47% of emergency contraceptive pill (ECP) users were under 25 years of age (9). In this study, nearly one-third of ECP users reported being unmarried. Research by Tamang et al. (5) conducted amongst 1,400 youth ages 15 to 24 (80.9% never married) in the Kathmandu Valley found that 35% of participants were sexually active. Amongst sexually active participants, only 17.5% reported condom use at first sexual intercourse, despite relatively high awareness of condoms as a safe sexual practice (92.7%). A further study by Khatiwada et al. (10), that analyzed data from the 2011 Demographic and Health Survey found that Nepalese youth (ages 15 to 24) have sexual experiences before marriage at an earlier age than seen in previous Demographic and Health Surveys despite traditional cultural and religious practices. Recent data from the 2022 Demographic and Health survey on testing rates for cervical cancer amongst women aged 15–49 found that only 6% received cervical cancer testing—7% of women who were tested for cervical cancer received a positive test result (11). These findings underscore the importance of focusing on the sexual health of this unmarried young population.

Moreover, 15.1% of Nepali men who were 15–24 years of age had sexual relations with an individual who was not their wife in the last 12 months with 41.3% reporting the use of a condom. More than three-quarters of young women (15–24) have heard of HIV or AIDS (80.2%) and 94.7% of men in the same age group. A small percentage (2.4%) of young women (15–19) had sexual relationships before the age of 15 and 2.1% of men. Although important health services such as abortion care have been legal in Nepal since 2002, data from the 2016 Demographic and Health Survey found that only 41% of female youth knew abortion was legal (12). A further study that analyzed data from abortion facilities across Nepal found that of 323,100 abortions, only 137,000 were completed legally, and 63,200 women were treated for abortion complications (13). Findings from a United Nations Population Fund (UNFPA) review of the literature on unmarried young people's (ages 10 to 24) sexual health behavior in Nepal between 2005 and 2015 highlighted the stigma from healthcare providers for sexual experiences before marriage that unmarried women face when seeking abortion care (8). To mitigate such barriers, the World Health Organization (WHO) recommends youth-friendly models of healthcare that offer equitable, accessible, acceptable, appropriate, and effective care to increase youth service access (14).

Given the importance of research on how to effectively deliver sexual health care to unmarried Nepalese young adults, the primary purpose of the current analysis is to identify correlates between Anderson's Behavioral Model of Health Services Use and sexual health service access amongst a sample of unmarried young adults living in Kathmandu, Nepal.

## Theoretical framework

**Figure 1:**
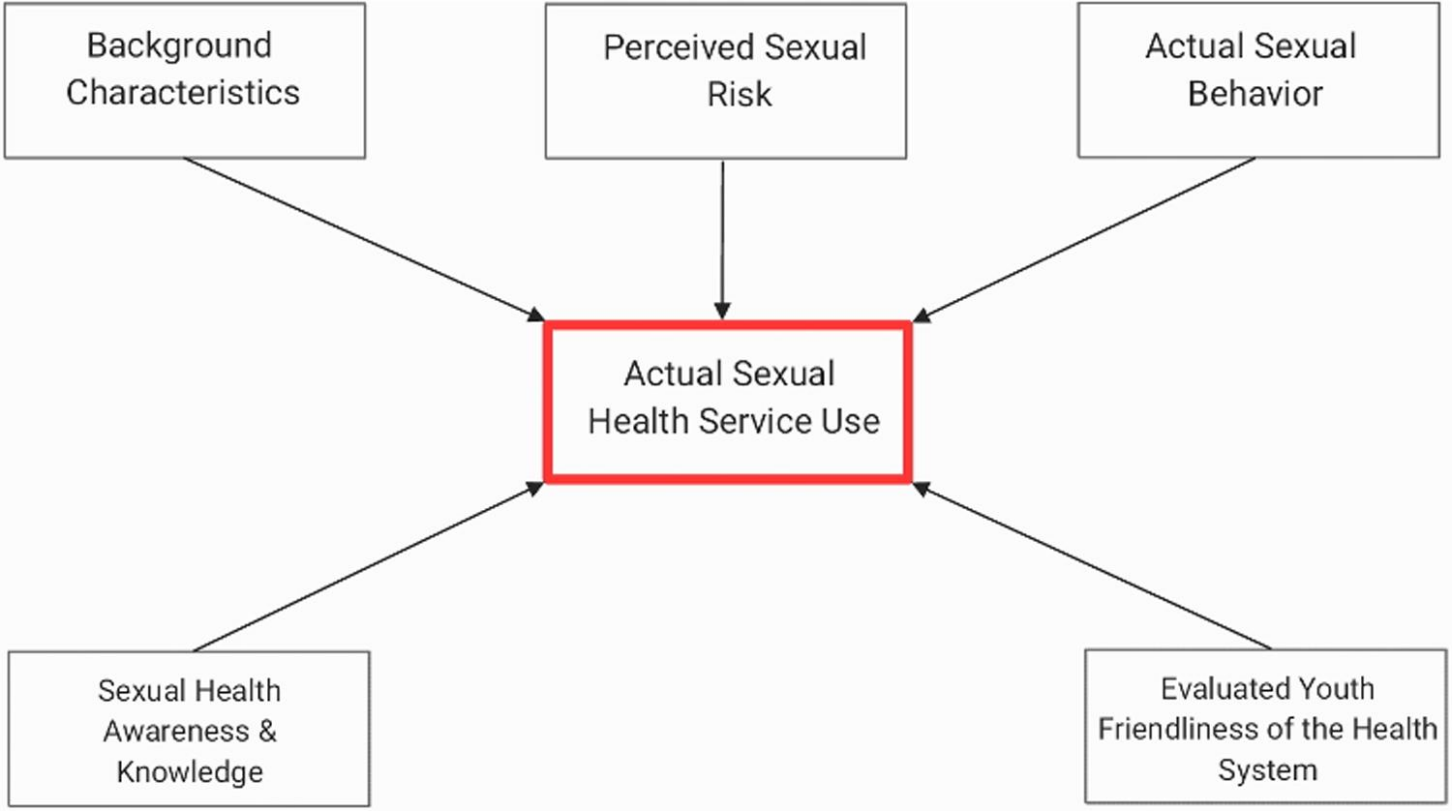
Andersen's behavioral model of health service use adapted framework.

Andersen's Behavioral Model of Health Service Use was a theoretical guide for this study (15). The model aims to predict and explain effective and efficient health service access. Adapting Andersen's model allows us to understand the most significant correlates of sexual health services, which these authors propose each carry equal weight. Figure 1 presents the adapted theoretical framework for this study, demonstrating the relationships among key constructs of interest. In this framework, the key explanatory constructs of interest are: (1) individual background characteristics (e.g., gender, religion, caste, sexual orientation, health behavior); (2) actual sexual behavior (e.g., sexual intercourse, condom use, dating history); (3) sexual health awareness and knowledge (e.g., abortion awareness, Human Papillomavirus [HPV] awareness, sexually transmitted infection (STI) awareness); (4) perceived sexual risk; and (5) evaluated youth friendliness of the health system. The outcome in this model is actual sexual health service use.

## Methods

### Study design, setting, and procedures

The study used a cross-sectional correlational design. Study participants were recruited from 10 wards in the Kathmandu Valley and the surrounding municipalities (Lalitpur, Khumaltar, Dhapakel, Bhaktapur, and Thumi). These wards and surrounding municipalities were selected due to the proximity of colleges and universities. Data were collected using a convenience sample method (16). Participants were approached using recruitment flyers distributed by research assistants to commuter students at three academic institutions. Study participation required a one-time in-person survey on a tablet. Eligibility criteria included being unmarried, residing in one of the selected recruitment areas, having lived in Kathmandu for at least one year, having visited a health clinic since the age of 15, not being currently pregnant, and being able to read and write in English (as the survey was administered in English). Participants had to have prior experiences visiting a health clinic to answer questions on their experiences with youth friendly healthcare.

Following written informed consent, participants completed a self-administered survey taking approximately 20 min. To minimize underreporting of sensitive sexual experiences, the survey was self-administered on a tablet in a private room. Information related to participant consent was gathered separately to reassure participants that their survey responses remained completely anonymous.

Participants received 100 Nepalese Rupees compensation for their time and effort (equivalent to US $1, or roughly the cost of lunch). Research assistants were present to complete informed consent, clarify survey content, and provide participant compensation. Institutional Review Board (IRB) approval for this study was obtained from the University of Pennsylvania IRB and Nepal Health Research Council.

### Study population

The study was powered to achieve 80% power at a *p* < .05 significance level. To achieve this, a sample of 200 participants would be appropriate. After data collection, a total of 271 individuals were screened for eligibility, 205 met eligibility criteria, and 203 agreed to complete the full survey with an overall response rate of 99%. The final sample for the regression analyses was 199 as four participants had missing data and were excluded from the analyses through case wise deletion. Of those who were not eligible to take part in the study, reasons included: incorrect age (*n* = 58), currently pregnant (*n* = 7), and one individual had not accessed a health facility since the age of 15. No individuals refused to be screened to take part in the study.

## Questionnaire

### Measures: independent variables

#### Background characteristics and health demographic characteristics

Basic background characteristics (e.g., age, gender, sexual orientation, caste, religion, educational level) and personal health behavior information included questions previously used on a structured questionnaire on sexual health designed by the World Health Organization and publicly available questions from the Nepal Demographic Health Survey (17, 18). Questions from these two surveys and all further questions were worded in a gender-neutral or sex/gender-inclusive manner so that all participants, regardless of gender, could respond to the questions. Key general health behaviors assessed were cigarette consumption and alcohol consumption in the past 12 months.

#### Sexual behaviour and related questions

To measure sexual behavior, participants responded to binary (yes/no) questions about whether they had ever engaged in sexual intercourse (defined as the insertion of the penis into the vagina or rectum), used a condom during their most recent intercourse, ever used a dating app, or ever had a boyfriend or girlfriend. Unwanted sexual experiences were assessed by asking participants if they had ever been touched on a part of their body without their consent. To assess forced sexual intercourse, participants were asked whether they had ever been forced to have sexual intercourse against their will by a stranger or by an older relative.

#### Sexual health and service awareness and knowledge benefit

To evaluate participants' sexual health and service awareness, participants were asked binary response (yes/no) questions about their awareness of HIV, STIs, condoms, oral contraceptive pills, injectable contraceptives, HPV, HPV vaccination, safe abortion facilities, and the legality of abortion. Participants were also asked two binary response (yes/no) questions on whether they thought they could improve their health by learning more about HIV as well as HPV.

#### Perceived sexual risk

Perceived sexual risk was measured using three items that asked participants about their perceived risk of getting HIV, getting an STI, or becoming pregnant. The three items on HIV and perceived STI risk came from an existing publicly available and tested Likert risk scale (19). An item on the perceived risk of pregnancy was included as an addition and followed the same format as the HIV and STI items. Participants could select their level of risk on a 5-point Likert item (0–4, i.e., no risk, small risk, 50/50 risk, high risk, very high risk). The three items were combined to give a score ranging from 0 to 12, with 12 indicating higher perceived sexual risk. The Cronbach's alpha for this scale in this study was 0.88.

#### Perceived youth friendliness of the health system

Participants were asked about their experiences visiting health clinics and hospitals since the age of 15 using ten 4-point (1–4) Likert item scale of ten questions to measure and address key elements of youth friendliness of the health system in Kathmandu. Summary scores ranged between 10 and 40, with higher scores indicating higher perceived youth friendliness of the health system. Participants were asked, for example, how much they agree or disagree with statements such as “the staff at the health clinic are friendly and respectful”. In this analysis, Cronbach's alpha was 0.72.

### Measures: outcome variable

#### Actual sexual health service use

Actual use of sexual health services was measured as a binary response (yes/no) question, where participants were asked whether they had visited a health clinic or doctor of any kind to receive services or information on various sexual and reproductive activities within the past year (i.e., contraception, pregnancy, abortion, or STI).

A review of all items with Nepali nurses and university students was conducted to ensure the cultural appropriateness of the terms used. The questionnaire was then pilot tested with a group of eleven young adults (not included in the final sample) to evaluate the clarity, comprehensibility, and acceptability of the questions.

## Data analysis

### Descriptive analysis

Descriptive analyses included means, medians, standard deviations, and contingency tables with proportions for categorical data. Baseline bivariate comparisons were based on logistic regression for each continuous and categorical predictor variable because the outcome variable was binary. Based on these comparisons, relevant variables at the *p* ≤ .20 level were treated as covariates in the subsequent logistic regression analysis (20). The threshold of *p* ≤ .20 at the bivariate stage was chosen to avoid prematurely excluding potential correlates of interest that could influence model interpretation. Demographic measures were treated as continuous or categorical, while sexual health service use in the past year was measured as a dichotomous outcome.

### Logistic regression analysis

Initially, all variables from the bivariate analysis that were significant at the *p* ≤ .20 level were included in the logistic model. We created a logistic regression model using a backward elimination approach to test correlates of actual use of sexual services. Using a backward elimination approach, we removed the variable with the highest *p*-value and re-ran the model using an iterative process. Because there were only four missing observations, we handled missing data by eliminating these four cases. Table 3 presents the final logistic regression model with correlates of actual sexual health service use in the past with significant variables from Andersen's Behavioral Model of Health Service Use.

Multicollinearity between independent variables was measured using the Variance Inflation Factor (VIF) statistic in addition to pairwise crosstabulation between correlate variables (21). The VIF among the correlate variables in both models ranged from 1.01 to 1.04, suggesting no strong evidence of multicollinearity. There is potential conceptual overlap between some variables, such as dating app use and a history of having a boyfriend or girlfriend. While both reflect relationship or partner-seeking behavior, they capture distinct constructs in this context: dating app use reflects engagement with technology-mediated relationships, whereas having a history of a boyfriend or girlfriend reflects relationship experiences more broadly, including in person interactions.

## Results

### Background characteristics and health demographic characteristics

A total of 93 males (45.8%) and 110 females (54.2%) took part in the study, with a mean participant age of 19.6 (SD = 1.7). When asked about sexual attraction, 88.6% identified that they were attracted to the opposite sex, 1.5% to the same sex, and 9.9% to both sexes equally. The participants were primarily studying to complete a bachelor's degree (57.6%), or in college (Grade 11 or 12; 36.5%). The most common religion was Hinduism (81.3%), followed by Buddhism (9.4%). The three most common castes were Newari (34.5%), Brahman (20.7%), and Chhetri (23.2%). Most participants (78.9%) perceived their financial situation to be good or very good. Approximately two-thirds of participants (64.8%) reported consuming alcohol in the past twelve months, and close to 30% had smoked cigarettes. Table 1 presents a full summary of the sociodemographic and health demographic variables.

**Table 1:** Socio-demographic background characteristic, health behavior, and actual sexual health service (SHS) use (*N* = 203).

Correlation variable	Total sample		Did not access SHS		Did access SHS		*P* *
	N/Mean	%/SD	N/Mean	%/SD	N/Mean	%/SD	
Age							
(Mean, SD)	19.66	1.69	19.70	1.67	19.39	1.83	0.36
Gender							
Male	93	45.8	74	80.4	18	19.6	0.03
Female	110	54.2	99	90.8	10	9.2	
Sexual Attraction							
Opposite sex	179	88.6	158	89.3	19	10.7	0.00
Same-sex	3	1.5	2	66.7	1	33.3	
Both equally	20	9.9	12	60.0	8	40.0	
Education Level							
Grade 8–10	2	1.0	2	100.0	0	0	0.01
Grade 10–12	29	14.3	25	89.3	3	10.7	
Grade 12	45	22.2	31	70.5	13	29.5	
Currently studying bachelors	117	57.6	105	89.7	12	10.3	
Bachelor's degree	10	4.9	10	100.0	0	0	
Religion							
Hindu	165	81.3	137	84.0	26	16.0	0.58
Buddhist	19	9.4	17	89.5	2	10.5	
Kirat	6	3.0	6	100.0	0	0	
Christian	8	3.9	8	100.0	0	0	
Atheist	4	2.0	4	100.0	0	0	
Other	1	0.5	1	100.0	0	0	
Caste							
Newari	70	34.5	62	91.2	6	8.8	0.70
Brahman	42	20.7	35	83.3	7	16.7	
Chhetri	47	23.2	38	80.9	9	19.1	
Tamang	4	2.0	4	100.0	0	0	
Janijanti	16	7.9	13	81.3	3	18.7	
Madhesi	8	3.9	7	87.5	1	12.5	
Other	16	7.9	14	87.5	2	12.5	
Spoken English							
Yes	168	83.2	145	87.4	21	12.6	0.22
No	34	16.8	27	79.4	7	20.6	
Time Lived in Kathmandu							
Less than one year	1	0.5	1	100.0	0	0	0.88
1–5 years	54	26.6	45	83.3	9	16.7	
6–10 years	20	9.9	17	85.0	3	15.0	
Greater than 10 years	128	63.1	110	87.3	16	12.7	
Employment Status							
Full time (Crete full-time part-time)	1	0.5	1	100.0	0	0	0.93
Part-time	19	9.4	17	89.5	2	10.5	
Seasonal	9	4.4	8	88.9	1	11.1	
Unemployed	174	85.7	147	85.5	25	14.5	
Student Status							
Yes	201	1.0	171	85.9	28	14.1	0.57
No	2	99.0	2	100.0	0	0	
Family Financial Situation							
Bad	5	21.2	5	100.0	0	0.0	0.58
Good	155	76.4	133	86.4	21	13.6	
Very Good	43	2.5	35	83.3	7	16.7	
Sexual & Health Behavior							
Alcohol Consumption							
Yes	129	64.8	106	82.8	22	17.2	0.10
No	70	35.2	63	91.3	6	8.7	
Cigarette Consumption							
Yes	60	29.6	48	80.0	12	20.0	0.11
No	143	70.4	125	88.7	16	11.4	
Sexual Intercourse							
Yes	74	36.6	58	80.6	14	19.4	0.10
No	128	63.4	114	89.1	14	10.9	
Condom use during last intercourse							
Yes	39	55.7	29	76.3	9	23.7	0.44
No	31	44.3	26	83.9	5	16.1	
Dating app use							
Yes	39	19.3	27	69.2	12	30.8	0.00
No	163	80.7	145	90.1	16	9.9	
Ever had a boy/girlfriend							
Yes	126	62.4	104	83.2	21	16.8	0.14
No	76	37.6	68	90.7	7	9.3	
Unwanted sexual contact							
Yes	83	40.9	71	85.5	12	14.5	0.86
No	120	59.1	102	86.4	16	13.6	
Forced sexual intercourse							
Yes	12	5.9	6	50.0	6	50.0	0.00
No	191	94.1	167	88.4	22	11.6	
Perceived Sexual Risk (0–12)							
(Mean, SD)	0.87	2.04	0.64	1.74	2.29	3.02	0.00
Youth Friendly Health Services Score (10–40)							
(Mean, SD)	28.89	3.35	28.62	3.32	30.44	3.09	0.00

* All *P* values were obtained from logistic regression. Percentages are column based.

### Sexual behaviour and related questions

Approximately 37% of participants reported engaging in sexual intercourse, and of those who reported engaging in sexual intercourse 55.7% reported using a condom during last intercourse. More males reported engaging in sexual intercourse than females (63.5% vs. 36.5%; *X^2^* = 14.35; *p* < .01). Close to two-thirds (62.4%) reported ever having a boyfriend or a girlfriend. In the study sample, 19.3% of the participants said they had used a dating app such as Tinder. More than a third (40.9%) of participants reported experiencing some form of unwanted sexual contact such as being touched on a part of their body that they did not want to be touched, and 5.9% reported experiencing forced sexual intercourse against their will. Although females reported more incidents of *unwanted* sexual contact (53.6% vs. 25.8%; *X^2^* = 16.15; *p* < .01), more males reported experiencing *forced* sexual intercourse than females (9.7% vs. 2.7%; *X^2^* = 4.38; *p* < .05). Table 2 presents a full summary of sexual behavior and related variables.

**Table 2:** Self-perceived sexual health awareness, the benefit of increased knowledge, and actual SHS use (*N* = 203).

Predictor variable	Sample size & percent		Did not access SHS		Did access SHS		*P* *
	*N*	%	*N*	%	*N*	%	
HIV Awareness							
Yes	185	91.1	157	85.8	26	14.2	0.72
No	18	18.9	16	88.9	2	11.1	
HIV Knowledge Health Benefit							
Yes	195	96.1	168	87.1	25	12.9	0.05
No	8	3.9	5	62.5	3	37.5	
STI Awareness							
Yes	146	71.9	124	85.5	21	14.5	0.72
No	57	28.1	49	87.5	7	12.5	
Condom Awareness							
Yes	201	99.0	172	86.4	27	13.6	0.14
No	2	1.0	1	50.0	1	50.0	
Oral Contraceptive Pill Awareness							
Yes	167	82.3	142	86.1	23	13.9	0.99
No	36	17.7	31	86.1	5	13.9	
Depo Provera Awareness							
Yes	67	33.0	53	79.1	14	20.9	0.04
No	136	67.0	120	89.6	14	10.4	
Legality of Abortion							
Yes	71	35.0	63	88.7	8	11.3	0.72
No	45	22.2	37	84.1	7	15.9	
Unsure	87	42.9	73	84.9	13	15.1	
Safe Abortion Location Awareness							
Yes	79	39.1	63	79.8	16	20.2	0.04
No	123	60.9	109	90.1	12	9.9	
HPV Awareness							
Yes	54	26.6	44	81.5	10	18.5	0.23
No	149	73.4	129	87.8	18	12.2	
HPV Vaccine Awareness							
Yes	40	19.9	29	72.5	11	27.5	0.00
No	161	80.1	142	89.3	17	10.7	
HPV Knowledge Health Benefit							
Yes	166	82.2	145	88.4	19	11.6	0.04
No	36	17.8	27	75.0	9	25.0	

* All *P* values were obtained from logistic regression. Percentages are column based.

### Perceived sexual risk

The majority (83.7%) of participants indicated no perceived risk of HIV, while 8.4% said they had a 50/50 risk of getting HIV. The majority (87.0%) of participants also indicated no perceived risk of getting someone pregnant or becoming pregnant. While 82.6% of participants said that they were at no risk of getting an STI, 7.0% said they had a 50/50 risk and 4.5% indicated a high or very high risk. The majority (76.6%) of participants' total perceived sexual risk score was 0 out of 12, which indicates no perceived sexual risk.

### Sexual health and service awareness and knowledge benefit

Awareness of STIs such as HIV was high (91.1%), while fewer (71.9%) were aware of STIs other than HIV, such as chlamydia. Nearly all participants (96.1%) indicated that they could improve their sexual health by learning more about HIV. Almost all (99%) were aware of the availability of condoms and 82.3% were aware of oral contraceptive pills. In contrast, awareness of injectable contraceptive methods such as Depo Provera was lower (33%).

Although abortion is legal in Nepal, only 35% of participants were aware that it is legal, while 65% thought that it was not legal or were unsure if it was legal. Less than half (39.1%) of participants knew of a safe place that someone could go to get an abortion. Awareness of human papillomavirus (HPV) was low, with only 26.6% of participants aware of HPV and 19.9% aware of the HPV vaccine. More than four in five participants (82.2%) indicated that they could benefit their health by learning more about HPV.

### Perceived youth friendliness of the health system

A large majority of participants (93.9%) agreed that it was easy to find a health clinic and 87.1% agreed that there is a health clinic close to where they live. When asked about clinic wait times, 60.9% said that their wait time was less than 30 min. Over half (58.6%) said that the health clinic was pleasant to sit in, while 41.4% said that it was not. Approximately three-quarters of participants (76.4%) agreed that the staff at the clinic were friendly and respectful, while 23.6% disagreed. A total of 90.2% of participants said that the information they received from the health clinic was easy to understand, helpful, and they felt that their privacy was respected. Most participants (87.2%) felt the information they shared with the health clinic staff would be kept confidential. Finally, 79.3% agreed that the cost of their clinic visits was affordable, while 22.7% of participants disagreed.

### Actual sexual health service Use

Overall, 13.9% of participants reported actual use of sexual health services in the past 12 months. Amongst participants who were ever sexually active (*n* = 74), 19.4% reported accessing a sexual health service compared to 10.9% for non-sexually active participants (19.4% vs. 10.9%; *X^2^* = 2.77; *p* < .1). Males reported higher actual service use compared to females (64.3% vs. 35.7%; *X^2^* = 4.49; *p* < .05).

### Bivariate analysis

Variables meeting the *p* ≤ .20 inclusion criterion included: gender (*p* = 0.03), sexual attraction (*p* < .01), education level (*p* = 0.01), alcohol consumption (*p* = 0.10), cigarette consumption (*p* = 0.11), sexual intercourse (*p* = 0.10), dating app use (*p* < .01), ever having a boy or girlfriend (*p* = 0.14), forced sexual intercourse (*p* < .01), perceived sexual risk (*p* < .01), perceived youth friendliness of the health system (*p* < .01), benefit of HIV knowledge (*p* = 0.05), condom awareness (*p* = 0.14), depo Provera awareness (*p* = 0.04), safe abortion location awareness (*p* = 0.04), HPV vaccine awareness (*p* < .01), and benefit of HPV knowledge (*p* = 0.04).

### Multivariable analysis

In a logistic regression analysis (Table 3) sexual health service use was regressed on the possible influential variables that were significant at the *p* ≤ 0.20 level based on bivariate analysis. Following a backwards elimination approach, in this exploratory logistic regression model, significant associations with sexual health service use were observed for perceived youth friendliness (OR: 1.19; 95%; CI: 1.01–1.39; *p* < .05), with same or both -sex sexual attraction (OR: 2.91; 95%; CI: 1.54–5.50; *p* < .01), cigarette consumption (OR: 3.05; 95%; CI: 1.04–8.94; *p* < .05), dating app use (OR: 3.59; 95%; CI: 1.25–10.3; *p* < .05), forced sexual intercourse (OR: 5.92; 95%; CI: 1.16–30.14; *p* < .05), perceived sexual risk (OR: 1.33; 95%; CI: 1.11–1.59; *p* < .01), and depo Provera awareness (OR: 2.81; 95%; CI: 1.01–7.81; *p* < .01). Gender, education level, alcohol consumption, sexual intercourse, ever having a boy or girlfriend, the benefit of HIV knowledge, condom awareness, safe abortion location awareness, the benefit of HPV knowledge, and HPV vaccine awareness were not found to be statistically significant.

**Table 3:** Final logistic regression model relating actual sexual health services with Andersen's behavioral model of health service Use significant variables (*n* = 199).

Regression variable^a^	Odds ratio	Confidence interval	*P*
Perceived Youth Friendly Health Service Score	1.19	1.01–1.39	0.03
Same or both -sex Sexual Attraction	2.91	1.54–5.50	0.00
Cigarette Consumption	3.05	1.04–8.94	0.04
Dating App Use	3.59	1.25–10.3	0.02
Forced Sexual Intercourse	5.92	1.16–30.14	0.03
Perceived Sexual Risk	1.33	1.11–1.59	0.00
Depo Provera Awareness	2.81	1.01–7.81	0.00

^a^ Pseudo R2 of 28.9%; ROC of 0.8.

## Discussion

This is one of the first studies to examine important theoretical factors associated with sexual health service use among unmarried young adults in Nepal. Our findings advance sexual health literature in Nepal by finding low perceived sexual risk amongst sexually active participants, high rates of unwanted and forced sexual contact, low awareness of the legality of abortion and where to find a safe abortion facility, low awareness of sexual health conditions such as HPV, and the importance of youth-friendly models for healthcare in a key population that is underrepresented in sexual health literature.

Perceived youth friendliness of the health system was positively associated with sexual health service use, underscoring the potential value of youth-friendly models of healthcare for unmarried young adults in Nepal. Dating app use was also positively associated with service use, suggesting that digital platforms may represent an important point of connection for sexual health promotion. In recent years, technology-based health interventions, particularly mHealth (22), have received attention as strategies to promote youth-friendly care. Given the widespread use of digital platforms in Nepal, integrating sexual health promotion into tech-based approaches may represent a feasible way to increase awareness of sexual health and connect unmarried young adults with healthcare providers.

Experiencing forced sexual intercourse was also associated with sexual health service use, with one in twenty participants (5.9%) reporting forced intercourse against their will. Interestingly, males reported higher rates of forced sexual intercourse than females, a finding that is inconsistent with existing literature. The finding that unmarried males reported higher rates of forced intercourse suggests the need for further research into sexual violence against unmarried youth in Nepal, as well as the potential clinical role of sexual health services in mitigating long-term effects of sexual violence.

Although unwanted sexual contact was more common (40.9%), it was not associated with sexual service use in this study. Prior research among female secondary school students in Nepal found high rates of sexual harassment but limited understanding of how to respond (23). Research on intimate partner violence among married women in Nepal has found high rates of physical or sexual violence that also led to higher odds of reporting unintended pregnancy (24–26). The literature on sexual violence amongst unmarried young adults in Nepal is more limited. It is also important to consider that social desirability bias may have led participants to underreport sexual activity, risky behaviors, or experiences with sexual violence, while overreporting socially acceptable responses such as willingness to learn about sexual health.

Several sexual health behaviors, knowledge gaps and low perceived sexual risk were identified as potential barriers to service use. First, awareness of HPV and HPV vaccination was low. Although HPV awareness was not significant in our multivariable analysis, it remains an important public health concern given HPV's causal relationship with cervical cancer (implicated in 99.7% of cervical carcinomas worldwide) (27). Prior research among married women in rural Nepal reported an HPV prevalence rate of 14.4% (28), yet HPV vaccination programs implemented in Nepal face barriers including cost and lack of awareness (28). Our findings that many unmarried young adults expressed willingness to learn more about HPV suggest a need to improve education and expand preventative options such as vaccination. In contrast, awareness of Depo-Provera was positively associated with sexual health service use, indicating that knowledge of specific contraceptive options may facilitate sexual healthcare seeking.

Second, awareness of abortion laws and safe abortion facilities was also low. This is relevant given Nepal's comparatively unrestrictive abortion laws in the South Asian region, which allow abortion up to 12 weeks for any indication and under specific circumstances thereafter (29). Despite this, many participants did not know where to access safe abortion services. This indicates the need for continued public education about both the legal framework and the availability of abortion services (30). Expanding education on contraception for unmarried young adults could also reduce unintended pregnancies and the demand for abortion care.

Finally, condom use among sexually active participants was low, and perceived sexual risk was minimal across the study population. While higher perceived risk scores were positively associated with higher reported service use, overall risk scores remained low, suggesting a limited perception of sexual risk. Prior research in Nepal also found low condom use among sexually active unmarried youth (48%) (31). The lack of perceived risk may increase the risk of unintended pregnancy, STIs, and HIV. Improving young adults' understanding of sexual risk is therefore clinically relevant to encourage both safer sexual practices and appropriate use of sexual health services.

### Implications and contribution statement

This study identified several factors associated with sexual health service use among unmarried young adults in Nepal. Perceived youth-friendliness of the health system, awareness of contraceptives such as Depo-Provera, use of online dating apps, experiences with forced sexual intercourse, and higher perceived sexual risk were positively associated with reported service use. For stakeholders, reinforcing youth-friendly care and supportive responses to sexual violence, while addressing gaps in knowledge, risk perception, and access, may help strengthen sexual healthcare service delivery. Future research using longitudinal or intervention-based designs is needed to test strategies that can effectively reduce barriers and enhance sexual health service use. Taking these steps could contribute to more effective sexual health service delivery for this population in Nepal.

### Study limitations

The findings may not be generalizable to all unmarried young adults in Kathmandu, because a convenience sample was used to recruit unmarried young adults primarily from colleges and universities in the Kathmandu area. In the future, it would be important for a larger study to recruit a more representative sample (e.g., increased variability in educational attainment) using a probability sampling technique to capture diverse perspectives. This would lead to a more representative sample with the inclusion of individuals without college or university backgrounds.

The study was only conducted in English which could have resulted in a loss of cultural meaning of words and the exclusion of the experiences of individuals who did not speak English. Of note, during recruitment no individual refused to take part in the eligibility screening process for this study, creating the potential for selection bias as those interested in the topic participated, while also suggesting that young adults in Nepal understand the importance of sexual health research and are willing to participate. Also, for those who experienced forced sex and unwanted sexual contact, we did not ask any demographic information about the perpetrator, such as age, gender, or relationship to the participant. Future research is needed that explores these contextual factors to inform prevention efforts.

## Data Availability

The raw data supporting the conclusions of this article will be made available by the authors, without undue reservation.
